# Early circulating tumor DNA dynamics as a pan‐tumor biomarker for long‐term clinical outcome in patients treated with durvalumab and tremelimumab

**DOI:** 10.1002/1878-0261.13349

**Published:** 2022-12-13

**Authors:** Maya Kansara, Neeru Bhardwaj, Subotheni Thavaneswaran, Chang Xu, Jessica K. Lee, Lo‐Bin Chang, Russell W. Madison, Frank Lin, Eugene Hsu, Vipul Kumar Patel, Alexey Aleshin, Geoffrey R. Oxnard, John Simes, Halla Nimeiri, David M. Thomas

**Affiliations:** ^1^ The Kinghorn Cancer Centre Garvan Institute of Medical Research Darlinghurst NSW Australia; ^2^ Faculty of Medicine, St. Vincent's Clinical School UNSW Sydney Kensington NSW Australia; ^3^ Foundation Medicine, Inc. Cambridge MA USA; ^4^ National Health and Medical Research Council Clinical Trials Centre University of Sydney NSW Australia; ^5^ Radiology Department St Vincent's Hospital Sydney NSW Australia; ^6^ Natera Austin TX USA

**Keywords:** biomarker, ctDNA, immune checkpoint inhibitor, pan tumor, treatment response monitoring

## Abstract

There is an urgent need to identify biomarkers of early response that can accurately predict the benefit of immune checkpoint inhibitors (ICI). Patients receiving durvalumab/tremelimumab had tumor samples sequenced before treatment (baseline) to identify variants for the design of a personalized circulating tumor (ctDNA) assay. ctDNA was assessed at baseline and at 4 and/or 8 weeks into treatment. Correlations between ctDNA changes to radiographic response and overall survival (OS) were made to assess potential clinical benefit. 35/40 patients (87.5%) had personalized ctDNA assays designed, and 29/35 (82.9%) had plasma available for baseline analysis, representing 16 unique solid tumor histologies. As early as 4 weeks after treatment, decline in ctDNA from baseline predicted improved OS (*P* = 0.0144; HR = 9.98) and ctDNA changes on treatment‐supported and refined radiographic response calls. ctDNA clearance at any time through week 8 identified complete responders by a median lead time of 11.5 months ahead of radiographic imaging. ctDNA response monitoring is emerging as a dynamic, personalized biomarker method that may predict survival outcomes in patients with diverse solid tumor histologies, complementing and sometimes preceding standard‐of‐care imaging assessments.

## Graphical abstract

We used a novel approach combining a tissue‐informed comprehensive genomic profiling assay with personalized blood‐based monitoring demonstrating that early ctDNA dynamics predict long‐term clinical outcomes in patients with different solid tumors treated with immune checkpoint inhibition. Molecular response as measured by ctDNA change from baseline represents an important emerging tool for assessing drug effect, complementary to imaging‐based response assessment.

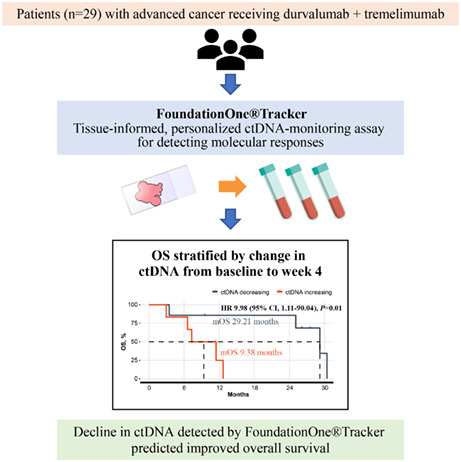

**Abbreviations**
cfDNA: cell‐free DNA
CGP: comprehensive genomic profiling
CR: complete response
ctDNA: circulating tumor
CTLA‐4: cytotoxic T lymphocyte antigen
ddPCR: droplet digital polymerase chain reaction
dsDNA: double‐stranded DNA
FFPE: formalin‐fixed, paraffin‐embedded
HS: high‐sensitivity
ICI: checkpoint inhibitor
IV: intravenously
MoST: Molecular Screening and Therapeutics
MSI‐H: microsatellite instability‐high
MTM/mL: mean tumor molecules per mL of plasma
NGS: next‐generation sequencing
OS: overall survival
PD: progressive disease
PD‐1: programmed cell death protein 1
PD‐L1: programmed death‐ligand 1
PFS: progression‐free survival
RANO: Response Assessment in Neuro‐Oncology
RCF: relative centrifugal force
RECIST: Response Evaluation Criteria in Solid Tumors
TIL: tumor‐infiltrating lymphocytes
TMB: tumor mutational burden
ΔctDNA: change in ctDNA

## 1 Introduction

Durvalumab and tremelimumab are humanized monoclonal antibodies that block binding to the inhibitory programmed death‐ligand 1 (PD‐L1) and cytotoxic T lymphocyte antigen (CTLA‐4) immune checkpoints, respectively [1, 2], and are being actively investigated for use as combination therapy in advanced cancers [3]. Despite the clinical potential of immunotherapy treatment to improve patient survival, to date, only a minority of patients experience long‐term benefit from treatment. Robust, predictive biomarkers are needed for monitoring response to immunotherapy [4], with utility across cancer types [5]. High tissue PD‐L1 expression, microsatellite instability‐high (MSI‐H), or tumor mutational burden (TMB) are biomarkers that, when present before treatment in certain tumor types, increase the likelihood of immunotherapy clinical benefit [6, 7, 8, 9]. However, they are static assessments and not reflective of changes that may occur while patients undergo treatment.

Circulating tumor DNA (ctDNA) has emerged as a potential biomarker that can be measured longitudinally to meet this clinical unmet need and may detect changes in tumor burden in real time. Moreover, studies have shown ctDNA to be predictive of patient survival outcomes and can be used to risk‐stratify patients, identifying those who are most likely to gain long‐term benefit from immunotherapy [10, 11, 12]. ctDNA monitoring of patients may provide rationale for clinicians to adapt therapy early to improve outcome or for discontinuing ineffective therapy to avoid unnecessary toxicity and financial burden. Currently, there are a range of approaches to detect and quantify ctDNA in patient blood samples, which are commonly based on tracking variants present in a patient's tumor tissue or in a fixed panel and assessed using next‐generation sequencing (NGS) and droplet digital polymerase chain reaction (ddPCR), among other modalities [13, 14, 15, 16].

Here, we evaluate the role of ctDNA dynamics to predict the long‐term clinical outcome in patients from a pan‐tumor, single‐arm phase II clinical trial (ACTRN12616001019493) of durvalumab plus tremelimumab and demonstrate the potential utility of a novel monitoring approach combining a clinical trial version of the comprehensive genomic profiling (CGP) assay FoundationOne®CDx with personalized ctDNA analysis using multiplexed amplicon NGS.

## 2 Materials and methods

### 2.1 Patients and study design

The Molecular Screening and Therapeutics (MoST) framework protocol is a multicenter, open‐label, phase IIa trial. Of the total 112 patients treated on trial, recruitment took place at separate time points for molecularly unselected modules (*n* = 64) and TMB‐enriched expanded modules (*n* = 48). TMB enrichment in the expanded cohort was considered relative to the original cohort. Two patients in the original cohort and 17 patients in the expanded cohort had an intermediate or high TMB, of whom 7 of 29 patients were in the subset with evaluable ctDNA. Patients were treated at four sites across Australia (St Vincent's [SSA: 2019/STE14511]; St George [SSA: 2021/STE02348]; Lifehouse [SSA: LH17.037]; Linear [no SSA available]). Ethical approval was granted by the St Vincent's Hospital Sydney Human Research Ethics Committee (approval number: HREC/16/SVH/23; after transition to REGIS in 2019: 2019/ETH11778) for all study sites. The study was performed in accordance with the Declaration of Helsinki. Central or institutional ethics and local research governance approval were obtained. All patients provided written informed consent for participation in this trial. An independent data and safety monitoring committee provided independent assessments of patient safety and trial progress. Key eligibility criteria included patients with pathologically confirmed, advanced, or metastatic solid cancer of any histological type; Eastern Cooperative Oncology Group performance status 0–1; measurable disease by Response Evaluation Criteria in Solid Tumors (recist) version 1.1; and adequate hepatic, renal, and marrow function. Patients were required to have failed, refused, be progressing on, or be intolerant of standard therapies for their tumor type and not previously treated with a programmed cell death protein 1 (PD‐1), PD‐L1, or CTLA‐4 inhibitor. Tumor and immune PD‐L1 immunohistochemistry were assessed following study enrollment.

### 2.2 Durvalumab plus tremelimumab treatment and clinical endpoints

All patients were treated with a fixed dose of durvalumab 1500 mg intravenously (IV) and tremelimumab 75 mg IV every 4 weeks for the first four cycles. After completion of four cycles, durvalumab alone was continued every 4 weeks for up to an additional nine doses. Treatment continued until disease progression or unmanageable toxicity occurred or a decision to stop therapy was made by the patient or clinician. Patients who achieved and maintained disease control through to the end of the 12‐month treatment period were permitted to restart combination treatment upon evidence of disease progression, as specified in the treatment protocol, as were patients who developed progressive disease (PD) within the first 12‐month treatment period, while receiving durvalumab monotherapy. Overall survival (OS) was defined as time from first dose date to the date of death, or the last date of follow‐up. Progression‐free survival (PFS) was defined as time from first dose date to the date of first progression event, death, or last follow‐up date, whichever occurred first. Progression was assessed based on recist version 1.1 or Response Assessment in Neuro‐Oncology (RANO) criteria on a predetermined schedule. For patients without target lesions, disposition was recorded as non–complete response (CR)/PD and PD as appropriate.

### 2.3 Immunohistochemistry

Formalin‐fixed, paraffin‐embedded (FFPE) archival tumor tissue was used for examination of tumor cell (PD‐L1) expression and tumor‐infiltrating lymphocytes (TIL). PD‐L1 immunohistochemistry was performed using the Ventana PD‐L1 (SP263) assay and cut‐offs for positivity set at 1%. A hematoxylin and eosin–stained slide was used for morphologic discrimination of lymphocytes in the tumor and its immediate periphery. TILs were quantified as the proportion of TILs out of total cells on a slide, and dichotomized as low and high using the median TIL percentage for the cohort (1%).

### 2.4 Human blood sample collection and processing

Venous blood ~10 mL was collected at baseline and at the beginning of cycles 2 and 3 during treatment by standard phlebotomy techniques into Streck [cell‐free DNA (cfDNA) BCT] tubes (Streck, La Vista, NE, USA). The time from blood collection to processing was 0–5 days (median: 2 days). Blood collected in Streck tubes was kept at room temperature before and during processing where samples were centrifuged at 1600 relative centrifugal force (RCF) for 15 min, after which plasma was transferred to a new tube and spun for an additional 10 min at 3200 *g*. Aliquots of plasma were stored at −80 °C before DNA extraction.

### 2.5 FFPE DNA extraction, quantification, and quality measurement

For each patient, 10 sections of 4 μm of FFPE tissue underwent DNA extraction. Tissue deparaffination was performed using deparaffinization solution (Qiagen, Germantown, MD, USA). DNA extraction was performed using AllPrep DNA/RNA kit (Qiagen). Quantification was performed using Qubit double‐stranded DNA (dsDNA) high‐sensitivity (HS) assay kit (Invitrogen, Waltham, MA, USA).

### 2.6 Tissue CGP to identify patient‐specific alterations

Comprehensive genomic profiling was performed retrospectively as described in Milbury et al. [17] using a clinical trial assay version of FoundationOne®CDx to identify patient‐specific alterations in the ctDNA cohort with the FoundationOne®Tracker assay. FoundationOne®Tracker is a tissue‐informed personalized ctDNA‐monitoring assay for detecting molecular responses for patients across a variety of tumor types [18, 19] (Fig. S1). Tissue CGP was performed in a Clinical Laboratory Improvement Amendments–certified, College of American Pathologists–accredited, New York State–approved laboratory (Foundation Medicine, Cambridge, MA, USA) on FFPE archival patient samples using a clinical trial assay version of the FoundationOne CDx assay, which allows detection of all four major classes of genomic alterations: substitutions, indels, copy number alterations and gene rearrangements/fusions in 324 genes along with complex genomic signatures such as MSI and TMB. Approval for this study, including a waiver of informed consent and a Health Insurance Portability and Accountability Act waiver of authorization, was obtained from the Western Institutional Review Board (Protocol No. 20152817). Sequencing was targeting > 500× median coverage with > 95% of exons at coverage > 100× using ≥ 50 ng genomic DNA. Briefly, extracted DNA was end‐repaired and A‐tailed and adapters were ligated and hybridization‐based captured, followed by sequencing on the Illumina^®^ HiSeq 4000 (Illumina, San Diego, CA, USA) [20]. TMB was calculated by counting the number of synonymous and non‐synonymous substitutions and indels present at 5% allele frequency or greater from 0.8 Mb of sequenced DNA, excluding driver and germline alterations [21]. The cutoffs for defining intermediate and high TMBs were 10 and 20 mutations/Mb, respectively. MSI was measured by evaluating the changes to 114 loci selected from a total set of 1897 that have adequate coverage. In a large training set of data from clinical specimens, we then used principal components analysis to project the 228‐dimension data onto a single dimension (the first principal component) that maximizes the data separation, producing an NGS‐based MSI score [22]. For CGP results that passed quality control metrics, a novel, proprietary algorithm was used to predict somatic probability of variants to minimize the selection of nontumor‐derived variants (germline, clonal hematopoiesis derived, sequencing artifacts) for input into the FoundationOne®Tracker assay.

### 2.7 Germline algorithm

A novel logistic regression model was implemented to predict the probability of a variant being somatic (somatic probability score) based on the difference between the observed variant allele frequency and the inferred expected germline variant allele frequency. This algorithm directly infers the expected germline allele frequency from known germline single nucleotide polymorphisms (SNPs) located on adjacent genomic region expected to have the same copy number with the variant in question. The algorithm then filters variants based on the somatic probability score, allele frequency and annotation, and compares the variants with databases of known SNPs and clonal hematopoiesis variants. Short variants with high somatic probability were selected and submitted for primer design for the monitoring assay.

### 2.8 Variant selection and primer design

To build the tumor‐specific ctDNA assay, up to 16 clonal SNVs from CGP results were selected using a proprietary algorithm (Natera, Inc.) with an aim to maximize the detectability of tumor DNA in patients' plasma. The selected SNVs were used to design PCR amplicons based on optimized design parameters, ensuring the uniqueness of the amplicon sequences in the human genome and the efficiency and compatibility of the amplicons.

### 2.9 Cell‐Free DNA extraction, library preparation, and plasma multiple‐PCR NGS workflow

FoundationOne Tracker was performed retrospectively on cfDNA extracted from 10 mL plasma. Each cfDNA sample was quantified by Quant‐iT High Sensitivity dsDNA Assay Kit (Invitrogen) following the manufacturer's instructions. Up to 66 ng (20 000 genome equivalents) of cfDNA from each plasma sample was used as input into library preparation. The cfDNA was end‐repaired, A‐tailed, and ligated with custom adapters, as previously described [23]. The purified ligation product was amplified and purified using Ampure XP beads (Agencourt/Beckman Coulter, Indianapolis, IN, USA). An aliquot of each library was used as the input into the patient‐specific 16‐plex PCR reaction. Samples were amplified using the patient‐specific assay and barcoded, followed by pooling the amplicons. Sequencing was performed on an Illumina HiSeq 2500 Rapid Run with 50 cycles of paired‐end reads using the Illumina Paired End v2 kit. All paired‐end reads were merged using pear software (Exelixis Lab, https://cme.h‐its.org/exelixis/web/software/pear/). Bases that do not match in forward and reverse reads or that have a low‐quality score were filtered out to exclude sequencing errors. Merged reads were mapped to the hg19 reference genome with Novoalign (http://www.novocraft.com/, accessed on 22 November 2021). Mapped sequencing reads went through a QC process to filter reads that are not on‐target PCR products. After the sequencing of the PCR products, the number of reads for each amplicon of a patient‐specific assay was determined. Individual targets have an average read depth of > 105 000×. Targets with more than 5000× sequencing coverage are included in the analyses.

### 2.10 Plasma variant calling

Based on the proprietary error model, a confidence score was calculated for each target variant detected using mutant and reference alleles depth of read, as previously described [23]. The presence of tumor DNA in the plasma was determined based on a validated combined confidence that takes all patient‐specific variants of the assay into account. In order to make a ctDNA‐positive call, it is critical to observe at least two SNVs above the selected confidence threshold [24].

### 2.11 ctDNA analysis statistics

To minimize bias, ctDNA measurements were conducted with blinding to clinical data, and patient treatment and clinical data collection were conducted without knowledge of ctDNA measurements. The primary analysis investigated the association between OS time and the change in ctDNA (ΔctDNA). Patients classified as nonshedder, meaning 0 MTM/mL detected at baseline and all on‐treatment time points, were not included in change from baseline analyses. For the 15 patients included in this analysis, ctDNA data were collected before treatment initiation (baseline) and then at either week 4 or 8 (or both) into treatment. The ΔctDNA level was categorized as ctDNA decreasing (ctDNA increasing) if ctDNA level measured by MTM/mL decreased (or increased) from baseline. Landmark analysis was performed at week 4 and week 8 to correlate ΔctDNA with OS. In the week 8 analysis, ctDNA level measured by MTM/mL at the latest time point (i.e., if week 8 data were available, week 8 was used; otherwise, week 4 was used) was compared with baseline to define ΔctDNA. Kaplan–Meier curves with median OS were used to estimate and visualize the survival distributions in ctDNA increasing and decreasing groups. Log‐rank test was used to evaluate the significance of the effect of ΔctDNA on OS. Cox proportional hazards regression modeling was used to estimate the hazard ratios. r version 4.2.1 (www.r‐project.org/) software was used for all statistical analyses and ggplot2 and survminer for visualization. Similar analyses were performed to evaluate how ΔctDNA associates with OS within the PD and CR/partial response (PR)/stable disease (SD) subgroups. These analyses were also performed to evaluate the association between OS and ctDNA clearance and immunotherapy tissue biomarkers.

## 3 Results

### 3.1 Detection of ctDNA using a tissue‐informed personalized assay in a pan‐tumor cohort

The MoST study enrolled 48 patients into the expansion cohort of the phase II clinical trial (ACTRN12616001019493) investigating durvalumab plus tremelimumab between November 2018 and November 2019. This trial permitted all pathologically confirmed advanced or metastatic solid cancers and enriched for those with intermediate or high TMB in the expansion cohort. The clinical results for the full study are reported [25]. In the expansion cohort, patients had a median PFS of 2.9 months (95% CI: 1.8–3.6), median OS of 11.9 months (95% CI: 11.0–14.8), and objective response rate of 14% [25].

**Fig. 1:**
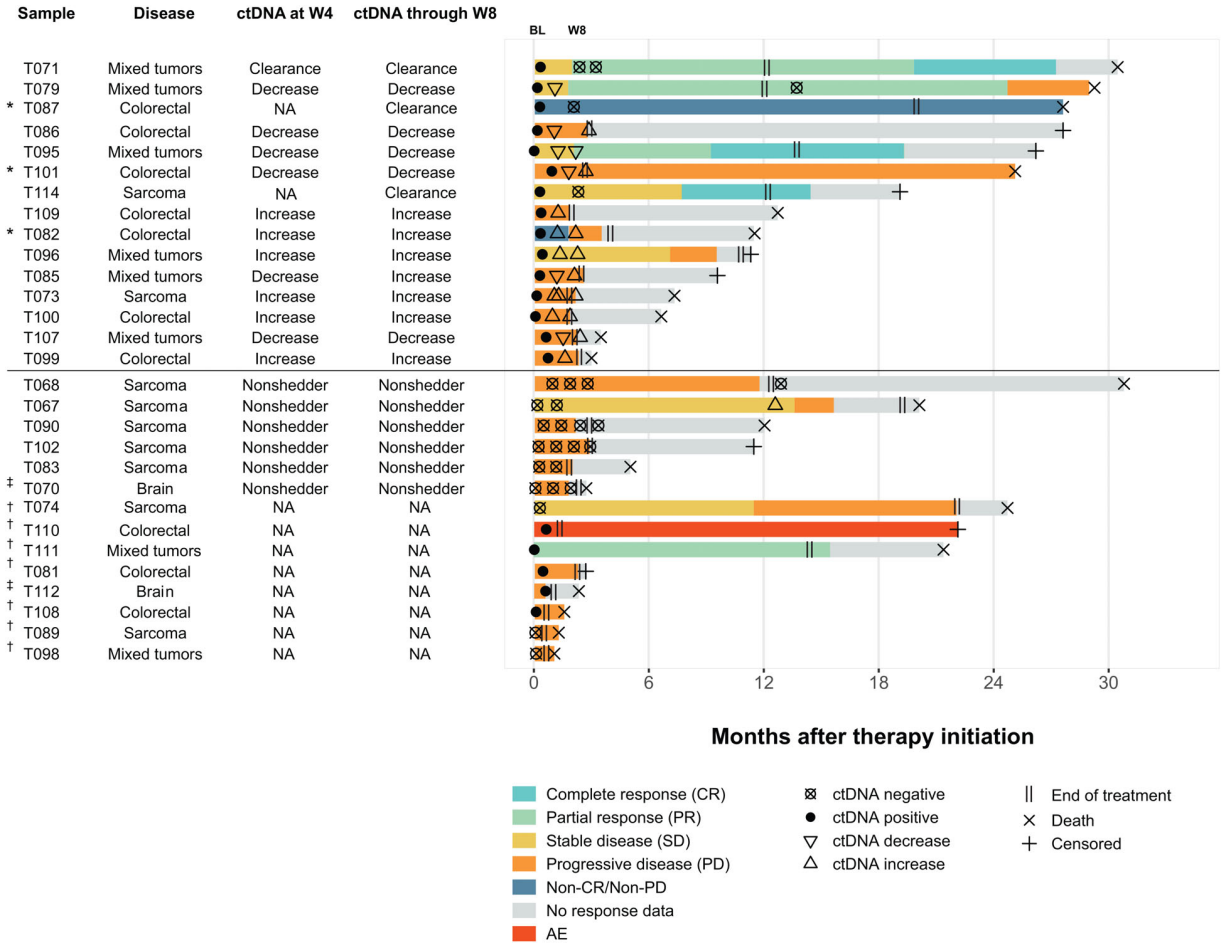
Swimmer plot showing ctDNA status, change in ctDNA from baseline, and clinical response. Twenty‐nine patients with available baseline ctDNA specimens are shown. The 21 patients with baseline and on‐treatment ctDNA samples are noted in the column “ctDNA at/through W4/W8.” Line segments are colored based on response assessed at each scan and the length of each segment corresponds to the time between scans. For the patients with no target lesions (NTL) by scan, clinical outcome was called “non‐CR/non‐PD” or “PD.” Patients with on‐treatment ctDNA changes from baseline are categorized as clearance (ctDNA decrease to 0 MTM/mL from baseline value), decrease (nonclearance), and increase. Nonshedder denotes six patients with ctDNA of 0 MTM/mL at baseline and on‐treatment assessment (excluded from analyses analyzing change from baseline). AE, adverse event; BL, baseline; ctDNA, circulating tumor DNA; MTM/mL, mean tumor molecules per mL of plasma; NA, sample not available for analysis or failed; NTL, no target lesions; RANO, Response Assessment in Neuro‐Oncology; RECIST, Response Evaluation Criteria in Solid Tumors; W4, week 4; W8, week 8. *Denotes patients with only NTL available for assessment. ^†^Denotes patients with no postbaseline scans available; for these patients, responses were determined based on clinical evidence. ^‡^Denotes glioma patients with RANO criteria for response. All other patients were assessed with RECIST.

The 48 patients enrolled in the expansion cohort had planned longitudinal plasma collection, with ctDNA analysis prespecified and analysis completed while blinded to clinical outcome. Forty of 48 patients from this study cohort were eligible for ctDNA analyses based on the remaining availability of baseline tumor tissue, essential for analysis of somatic tumor variants to follow in patient plasma. CGP using a clinical trial assay version of FoundationOne®CDx was performed on archival tissue specimens to create a personalized panel of two to 16 somatic variants to monitor patient tumor response to durvalumab plus tremelimumab (Fig. S1). Of these 40 patients, 35 (87.5%) had ≥ 2 trackable somatic variants identified from tissue baseline samples with successful primer design, and 29 of 35 had pretreatment plasma available for analysis and thus were included in the baseline analysis (Fig. 1; Fig. S2). The median time between tissue collection date and baseline ctDNA assessment was 404 days for these 29 patients (range, 39–1766 days). These 29 patients represented the following four major disease groups: colorectal cancer (*n* = 10), sarcoma (*n* = 9), mixed solid tumors (*n* = 8), and glioma (*n* = 2), corresponding to 16 unique cancer histologies (Table S1), and had a median PFS of 1.8 months and OS of 20.1 months.

**Fig. 2:**
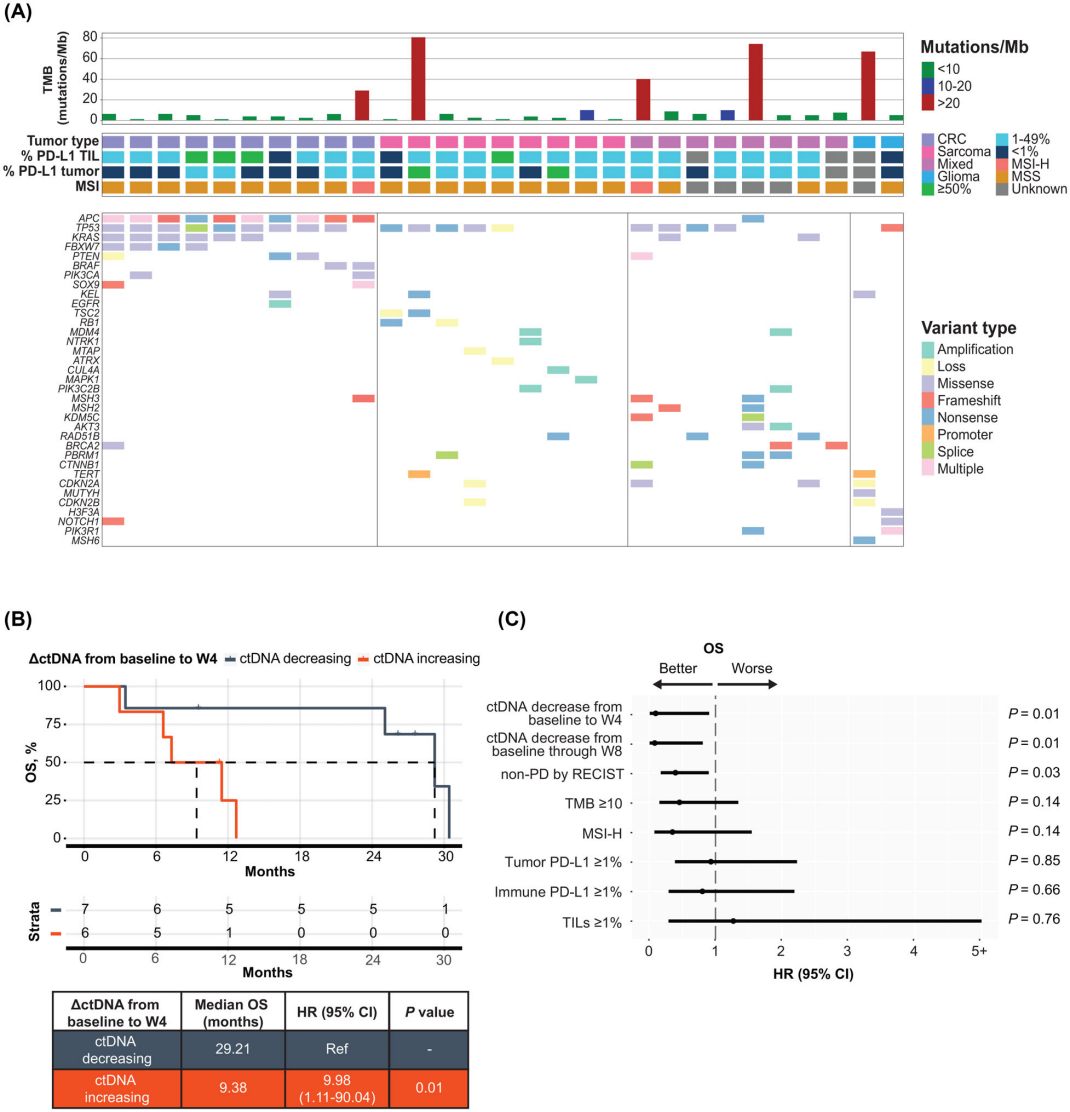
Change in ctDNA from baseline to W4 is strongly correlated with OS. (A) Oncoprint showing the landscape of clinically relevant baseline genomic alterations determined from tissue profiling of the 29 patients included in the baseline ctDNA analysis, as well as tumor type, %PD‐L1 staining (TIL, tumor) and MSI status. (B) Kaplan–Meier curve of OS for patients stratified by increasing versus decreasing ctDNA from baseline to W4. (C) Forest plot depicting unadjusted hazard ratios and 95% confidence intervals from univariate analyses. ΔctDNA, change in circulating tumor DNA; CI, confidence interval; CRC, colorectal cancer; ctDNA, circulating tumor DNA; MSI, microsatellite instability; MSI‐H, microsatellite instability‐high; MSS, microsatellite stability; OS, overall survival; PD‐L1, programmed cell death‐ligand 1; ref, reference; TIL, tumor‐infiltrating lymphocyte; TMB, tumor mutational burden; W4, week 4; W8, week 8.

A CGP analysis of baseline tissue from the 29 patients with pretreatment plasma samples revealed distinct genomics per disease subgroup, with only *TP53* observed as a recurrent oncogenic alteration across subgroups (65.5% overall prevalence; Fig. 2). Clinically relevant alterations detected varied on an individual basis consistent with the diversity of the cohort. Seven patient samples (24%) were evaluated to have TMB ≥ 10 in the ctDNA cohort, 2 (6.9%; 1 colorectal cancer, 1 prostate), of which were also MSI‐H. All colorectal cancer tumors harbored alterations in the WNT pathway regulator *APC*; 6 (60%) were *KRAS* mutated, which were mutually exclusive from *BRAF* V600E mutations [*n* = 2 (20%)]. *TSC2* mutations, which are common across several sarcoma types, were detected in 2 (22%) of the sarcoma cases. Given the varied tumor histology in the analyzed cohort, other recurrent oncogenic alterations were not observed.

Overall, baseline detection of ctDNA was achieved in 20 of 29 patients (69%; Fig. S3), and the rate of detection varied among the four disease groups. Baseline, week 4 and week 8 MTM/mL values, number of variants tracked per sample, as well as TMB and PD‐L1 are listed in Table S1. Higher ctDNA shed into blood was anticipated in the colorectal and mixed solid tumor disease groups based on previous analyses [16, 26]; accordingly, in these groups, 100% and 87.5% of successfully assayed samples, respectively, had detectable baseline ctDNA, compared with the lower shed groups of glioma (50%) and sarcoma (28.6%). The median quantitative measure of baseline ctDNA in detectable samples similarly varied as follows: colorectal (325 MTM/mL), mixed solid tumor (135 MTM/mL), glioma (0.20 MTM/mL), and sarcoma (0 MTM/mL; Fig. S3). Notably, there was not a significant association between baseline recist version 1.1 target lesion measurements and quantitative ctDNA measurements in the ctDNA baseline analysis population (*P* = 0.31; Fig. S3).

### 3.2 Early ΔctDNA levels from baseline predicts for long‐term survival

Fifteen patients had ctDNA measurements at both baseline and postbaseline (through week 8) available for ctDNA dynamics analyses. Of the 15 patients, 13 (87%) were evaluable as early as 4 weeks after initiation of durvalumab plus tremelimumab treatment, corresponding to completion of one cycle of doublet immune checkpoint inhibitors (ICI) in patients from three of four disease groups (sarcoma, colorectal cancer, and mixed solid tumor). Plasma samples from 7 out of 13 (54%) evaluable patients at week 4 had a decrease in ctDNA from baseline, which was strongly correlated with long‐term survival benefit as compared with an increase in ctDNA over the same time period (Fig. 2; median OS = 29.2 vs. 9.4 months; *P* = 0.0144; HR = 9.98). An equivalent and consistent result was obtained in plasma samples analyzed for ctDNA, collected through to 8 weeks, after completion of up to two cycles of therapy (Fig. S4; median OS = 29.2 vs. 11.5 months; *P* = 0.0105; HR = 11.6), illustrating the value of an early ctDNA assessment.

To assess the strength of this correlation, univariate analyses across the entire baseline ctDNA cohort of patients were performed. ctDNA decreases at week 4 or through week 8 were the only significant genomic biomarkers for survival benefit in this heterogeneous cohort of cancer histologies (Fig. 2C; *P* = 0.01). Other known immunotherapy biomarkers like MSI‐H, TMB ≥ 10, and tumor and immune PD‐L1 immunohistochemistry were not significantly associated with survival in this cohort of patients. Taken together, these data illustrate the potential of early, dynamic quantitative ΔctDNA to predict for long‐term benefit in advanced solid tumors.

### 3.3 ctDNA response can stratify RECIST groups and identify patients with highly favorable survival

**Fig. 3:**
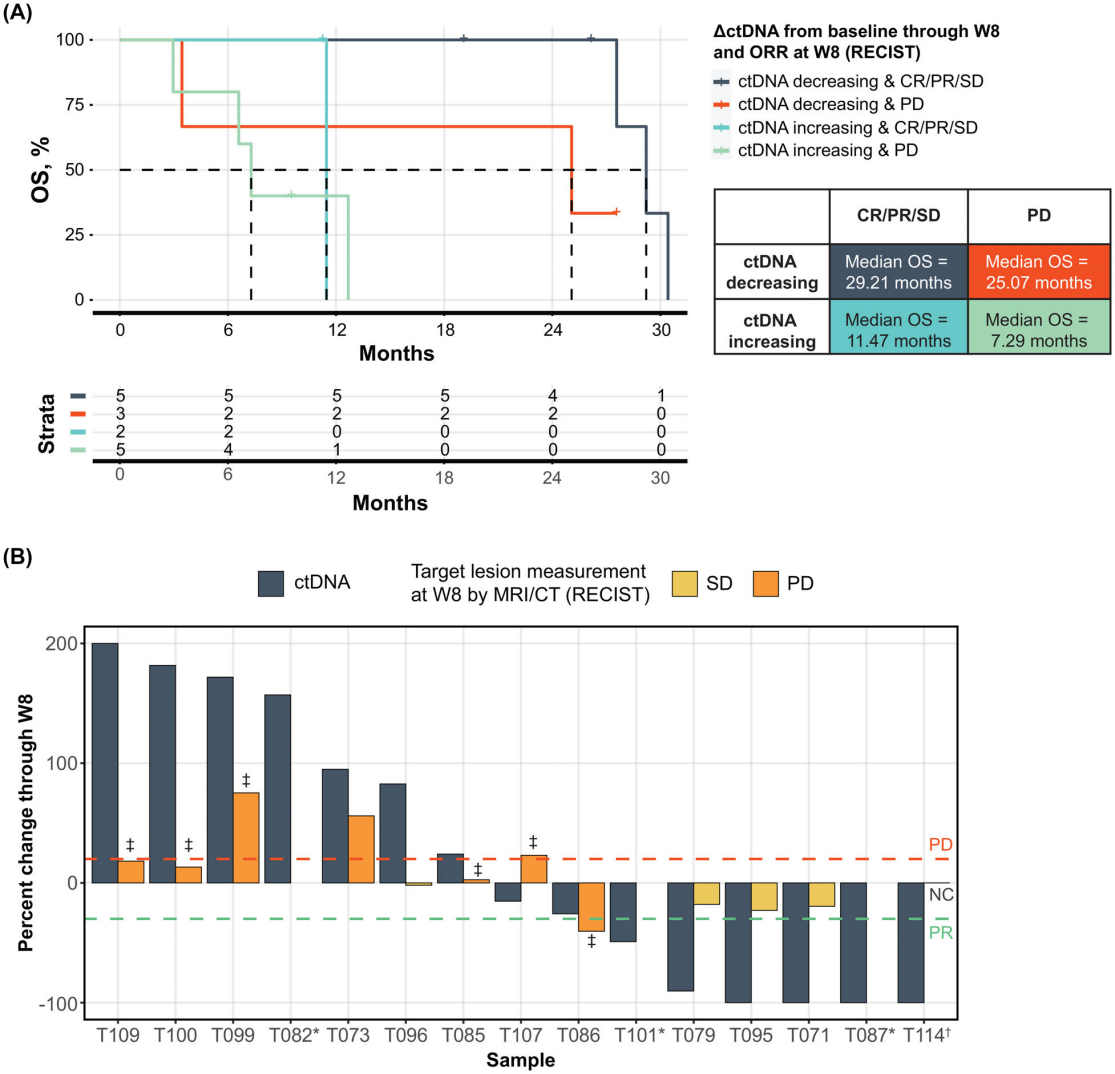
ctDNA response can stratify RECIST groups and identify patients with highly favorable survival. (A) Kaplan–Meier curve of OS for patients stratified by their ctDNA change through W8 and RECIST response at W8 for 15 patients with available baseline and on‐treatment ctDNA samples. (B) Waterfall plot of ctDNA change and RECIST response through W8. Three patients (T071, T087, T114) experienced ctDNA clearance by W8. *Patients T082, T101, and T087 did not have a target lesion available for RECIST assessment. ^†^T114 had no change (NC) from baseline TL assessment. ^‡^Denotes appearance of new lesions. PD line at +20% and PR line at −30% change from baseline. ΔctDNA, change in circulating tumor DNA; CR, complete response; CT, computed tomography; ctDNA, circulating tumor DNA; ORR, overall response rate; OS, overall survival; PD, progressive disease; PR, partial response; RECIST, Response Evaluation Criteria in Solid Tumors; SD, stable disease; W8, week 8.

Early ctDNA dynamics have the potential to distinguish true progressors on immunotherapy from pseudoprogression [27]. Using best overall response (recist version 1.1), ctDNA changes through week 8 were able to stratify best response groupings (Fig. 3). Patients with ctDNA decreases through week 8 and CR, PR, or SD as best response had the highest OS. Conversely, patients with ctDNA increase through week 8 and a best response of PD had the lowest OS (median OS = 29.21 vs. 7.29 months; *P* = 0.007). These two scenarios, in which changes in the ctDNA signal and best response align, are representative of how ctDNA and scan response measurements may validate each other to increase confidence regarding patient response to treatment. Interestingly, regardless of eventual best response, patients with ctDNA decrease through week 8 had prolonged OS compared with those with ctDNA increase. In the seven patients with CR, PR, or SD, those with ctDNA decrease (Fig. 3; dark blue, *n* = 5) had improved survival over others with ctDNA increase (light blue, *n* = 2) who were in the same radiographic response group (median OS = 29.21 months vs. 11.46 months; *P* = 0.0253). Of the eight patients with the best response of PD, three had a decrease in ctDNA by week 8 (Fig. 3; orange, *n* = 3). Even with early treatment discontinuation at time of PD, these patients sustained a long‐term benefit (median OS = 25.07 months). Overall, early ctDNA dynamics corresponded to changes in tumor measurements (Fig. S5).

At week 8, patients were assessed by recist version 1.1 criteria for their first follow‐up imaging on therapy. ctDNA changes from baseline assessed through week 8 were able to confirm or refine these time‐matched response assessments (Fig. 3B). In four of the eight patients with PD, an increase in ctDNA was detectable by week 4 (Fig. S6). In three patients assessed as having PD at week 8, a decrease in ctDNA was instead detected, which was reflective of the prolonged survival time in two of three patients (T086, T101; OS = 26.32 and 25.07 months, respectively). These two patients were categorized as PD due to the appearance of new bone and lung lesions upon evaluation; the target lesions for T086 were evaluated as PR, and T101 was unable to be assessed by recist version 1.1 criteria because of lack of qualifying target lesions. These patients may represent a setting in which ctDNA assessment can aid in interpretation of challenging or mixed radiologic response [28].

In addition to the eight patients with radiographic PD determined at week 8, seven patients were evaluated as SD or non‐CR/PD (Fig. 3; Fig. S5), all of whom went on to achieve a best response of CR, PR, SD, or non‐CR/PD. Two of these seven patients, with biliary or colorectal cancer, had an increase in ctDNA through week 8, as well as a shorter OS of 3.5 and 9.1 months, respectively. This result suggests that these two patients may not have derived benefit from the trial drug beyond their week 8 ctDNA assessment. Five of these seven patients had a decrease in ctDNA through week 8, three of whom achieved ctDNA clearance by week 8, and two who achieved reductions of 90.3% to 99.9% from baseline ctDNA levels, indicating potential long‐term benefit of therapy. These results illustrate how ctDNA assessment can be used in addition to standard‐of‐care radiologic assessment to provide an enhanced, dynamic view of patient response to therapy, particularly in cases of SD or non‐CR/PD.

**Fig. 4:**
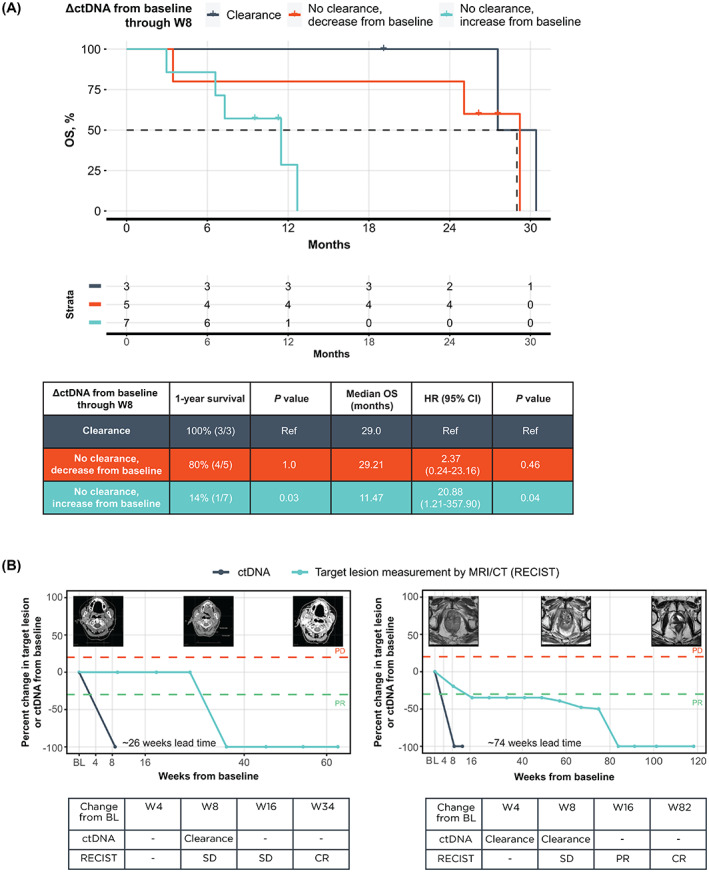
ctDNA clearance identifies patients with highly favorable survival. (A) Kaplan–Meier curve of OS for patients stratified by clearance and change in ctDNA from baseline through W8. (B) Two patients had ctDNA clearance followed by a complete RECIST response. ΔctDNA, change in circulating tumor DNA; BL, baseline; CI, confidence interval; CR, complete response; CT, computed tomography; ctDNA, circulating tumor DNA; OS, overall survival; PD, progressive disease; PR, partial response; RECIST, Response Evaluation Criteria in Solid Tumors; ref, reference; SD, stable disease; W4, week 4; W8, week 8; W16, week 16; W82, week 82.

Clearance of ctDNA on treatment has previously been linked to highly favorable patient outcomes [10, 11, 29]. Patients with ctDNA clearance had similar OS benefits as patients who had a decrease (nonclearance; range: −15.3% to −99.9%) in ctDNA levels through week 8, and significantly longer OS than those patients with an increase (range: +24.1 to +386.9%) in ctDNA through week 8 (Fig. 4; median OS = 29.0 vs. 11.5 months; *P* = 0.03). Two of three patients with ctDNA clearance through week 8 had measurable target lesions by recist version 1.1, and both achieved a best response of CR. Overall, there were three patients with CR in this subset of patients, with two achieving ctDNA clearance and the third achieving a 99.9% ctDNA reduction by week 8 on therapy. Both ctDNA clearance patients with CR were assessed as having SD at their week 8 radiographic assessment. Remarkably, ctDNA clearance in these patients preceded radiographic CR by a median of 11.5 months (50 weeks), and any radiographic response (CR or PR) by 3.8 months (16.5 weeks), exhibiting a lead time advantage in the use of ctDNA for assessment in these two patients.

## 4 Discussion

In this study evaluating durvalumab plus tremelimumab, we aimed to identify a robust response biomarker that could predict for long‐term survival benefit across the histologic and genomic diversity of the cohort. Moreover, a tumor‐agnostic biomarker can be used in the design of pan‐tumor trials to assess response across heterogenous cohorts of patients.

We demonstrated that a reduction in ctDNA as early as 4 weeks into immunotherapy treatment (one cycle of durvalumab plus tremelimumab) was a predictor for long‐term survival benefit in this cohort, with a median OS 29.21 versus 9.4 months in the ctDNA decrease and ctDNA increase groups, respectively. In addition, ctDNA clearance by week 8 was seen in two of three cases of radiologic CR in this pan‐tumor population. Compared with the standard immune biomarkers like PD‐L1, TMB, and MSI‐H, which are assessed before treatment, longitudinal assessment of ctDNA dynamics provides an accessible view into tumor response to therapy, as seen by the correlation between changes in ctDNA levels from baseline to time‐matched changes in tumor sum of the longest diameter measurements (Fig. S5B).

ctDNA dynamics add important data to help with evaluation of patient response to treatment. In our study, ctDNA dynamics were able to refine and, in some cases, precede radiographic response assessments, as seen in the two patients who achieved ctDNA clearance on treatment. In the five of 15 cases (33%) in which the ΔctDNA through week 8 was discordant with patient best response by recist version 1.1, the ctDNA response groups more strongly corresponded to survival benefit (Fig. 3A). Three of eight patients with radiologic PD at week 8 discontinued ICI therapy after their PD assessment but were retrospectively found to have decreasing ctDNA, suggestive of a molecular response. In such cases, ctDNA dynamics may provide complementary information to scans, particularly when the disease is radiologically difficult to evaluate. In addition, at week 8, two of seven patients evaluated as having SD or non‐CR/PD by imaging were retrospectively found to have increasing ctDNA at their time‐matched ctDNA assessment. The increase in ctDNA levels on treatment could identify patients who may not benefit from treatment with ICI and may exhibit poorer clinical course. Additional studies of patients with advanced‐stage cancers receiving ICIs have shown that ctDNA changes could predict clinical outcomes across different tumor types [10, 14, 16], further supporting the use of ctDNA dynamic changes as a response biomarker. Larger studies confirming the applicability of ctDNA dynamics as a response biomarker in a pan‐tumor setting are warranted.

Based on our results, early ctDNA dynamic changes could complement or refine radiologic assessments, especially in cases of mixed or unevaluable radiologic response to treatment. Notably, three of 15 patients with on‐treatment ctDNA results did not have measurable target lesions according to recist version 1.1 criteria but were able to be evaluated by ctDNA analysis, providing additional potential benefit to patients with limitations in imaging. With additional research, ctDNA dynamics could be extended to enable clinicians to adapt therapy based on ctDNA dynamics. For example, in the context of a rise in ctDNA on therapy, therapy intensification (e.g., dose, frequency) or a change in line of therapy could be considered, whereas a decrease or clearance in ctDNA may enable treatment de‐intensification or cessation. In both cases, this could allow patients to avoid the medical and financial toxicities associated with ineffective or unnecessary treatment. Owing to the intensity of care, out‐of‐pocket costs can often be high for patients with advanced‐stage cancer, a financial burden disproportionately placed on patients who are uninsured [30, 31]. Additionally, with the rising costs of cancer care, avoiding unnecessary therapy cycles is critically important for both individual patients and society [32, 33]. However, the promise of a ctDNA response adaptive paradigm must be evaluated prospectively in clinical studies, building upon recent advances supporting ctDNA detection to guide a therapy switch [34].

The ctDNA response monitoring assay used in this study is a novel approach personalized to the patient using a clinically informative baseline tissue CGP, dependent upon tissue availability. The promise of this type of approach is that it could enable clinicians to select precision‐based targeted therapy using the baseline CGP and add on the option to monitor ctDNA dynamics for their patients using a minimally invasive blood draw that can be repeated longitudinally to assess ctDNA response over time. We did not study serum tumor markers (e.g., carcinoembryonic antigen) in this analysis, which could be tracked to assess treatment response in addition to radiology. However, such assays are not an option for all tumor types and can be associated with a long half‐life that delays dynamic response assessment and varying levels of clinical sensitivity and specificity [35, 36, 37, 38]. Here, we showed the value of this ctDNA dynamics assessment to predict for OS across a diverse patient population, illustrating potentially broader utility as a complement to imaging techniques.

Our analysis of ctDNA dynamics was limited by a few factors. ctDNA changes on therapy were assessed in a retrospective manner in a single‐arm study, thus precluding comparison with a control group. The patients eligible for ctDNA analysis were from one cohort of the full MoST durvalumab plus tremelimumab study, representing a smaller patient population. Residual tumor DNA and plasma samples were not available for all patients at all on‐treatment time points, and there was additional attrition because of plasma volumes being below the recommended range (< 4 mL), resulting in an increase in sample failures (Fig. S2) and a smaller population (*n* = 15) with longitudinal ctDNA analyses. We did not further define clinically informative percent changes in ctDNA from baseline as this analysis would be limited by sample size; however, further assessment to distinguish ctDNA dynamics due to tumor response from natural variation is warranted. Additionally, even though ctDNA decrease trended with better PFS, the association was not statistically significant given the limited sample size in this study. The practice of ctDNA monitoring will benefit from continued investigation into the relationship between the magnitude and kinetics of ctDNA changes on therapy and patient outcomes in larger populations to inform a more prescriptive approach to patient management.

## 5 Conclusions

Overall, this study demonstrates that a decrease in ctDNA levels evaluated as early as 4 weeks into treatment using a novel assay was associated with long‐term clinical outcomes in a pan‐tumor cohort of patients with durvalumab plus tremelimumab treatment. The decrease in ctDNA on treatment could relate to treatment response to immunotherapy or by identifying those patients with a better prognosis. The mix of solid cancer types in this study are representative of real‐world clinical practice, highlighting the potential for ctDNA monitoring in a standard‐of‐care setting to help identify patients most likely to derive long‐term clinical benefit. Prospective validation of this biomarker in a larger study should be performed to confirm this finding and extend its use in optimizing treatment duration, which may change practice in the setting of doublet ICI, and applied to other therapeutic approaches, as is currently under investigation.

## Conflict of interest

MK: No competing interests; ST: No competing interests; DMT: CEO of Omico, and have received consultancies and research funding from Roche; FL: No competing interests; EH: No competing interests; JS: No competing interests; VKP and AA: Employees of Natera, Inc. with stock or options to own stock in the company; NB, CX, JKL, L‐BC, RWM, GRO: Employees of Foundation Medicine, Inc., a wholly owned subsidiary of Roche, and report stock ownership in Roche; HN: Previous employee of Foundation Medicine, Inc., a wholly owned subsidiary of Roche, and report stock ownership in Roche.

## Author contributions

MK: conceptualization, data curation, formal analysis, project administration, writing original draft, writing‐review and editing; NB: conceptualization, formal analysis, supervision, writing‐original draft, writing‐review & editing; ST: data curation, formal analysis, project administration, writing‐review and editing; CX: formal analysis, methodology, writing‐review & editing; JKL: data curation, formal analysis, visualization, writing‐review & editing; L‐BC: data curation, formal analysis, visualization, writing‐original draft, writing‐review & editing; RWM: data curation, formal analysis, methodology, visualization, writing‐review & editing; FL: data curation, formal analysis; EH: data curation, formal analysis; VKP: data curation, project administration, writing‐review & editing; AA: conceptualization, supervision, writing‐review & editing; GRO: conceptualization, supervision, writing‐review & editing; JS: formal analysis, writing‐ review and editing; HN: conceptualization, supervision, writing‐original draft, writing‐review & editing; DMT: conceptualization, supervision, writing‐original draft, writing‐review & editing.

### Peer review

The peer review history for this article is available at https://publons.com/publon/10.1002/1878‐0261.13349.

## Supporting information


**Fig. S1.** FoundationOne Tracker workflow diagram.
**Fig. S2.** Consort diagram.
**Fig. S3.** Baseline ctDNA level varies by disease type and trends with OS.
**Fig. S4.** Change in ctDNA from baseline through W8 is strongly correlated with OS.
**Fig. S5.** Change in ctDNA from baseline through W8 is correlated with change in target lesion size and survival.
**Fig. S6.** Relationship between ctDNA change and clinical response.

**Table S1.** Unique cancer histologies and other sample characteristics of ctDNA analysis cohort.

## Data Availability

All data relevant to the study are included in the article or uploaded in the Supporting Information.
