# Supplementary material for: Early circulating tumor DNA dynamics as a pan‐tumor biomarker for long‐term clinical outcome in patients treated with durvalumab and tremelimumab
Source: Mol Oncol. 2022 Dec 13;17(2):298–311. doi: 10.1002/1878-0261.13349 (PMC9892824; doi:10.1002/1878-0261.13349)

**Supplementary Figure S1.**

Workflow of FoundationOne Tracker. Abbreviations: CTA, clinical trial assay; MTM/mL, mean tumor molecules per mL of plasma.

**
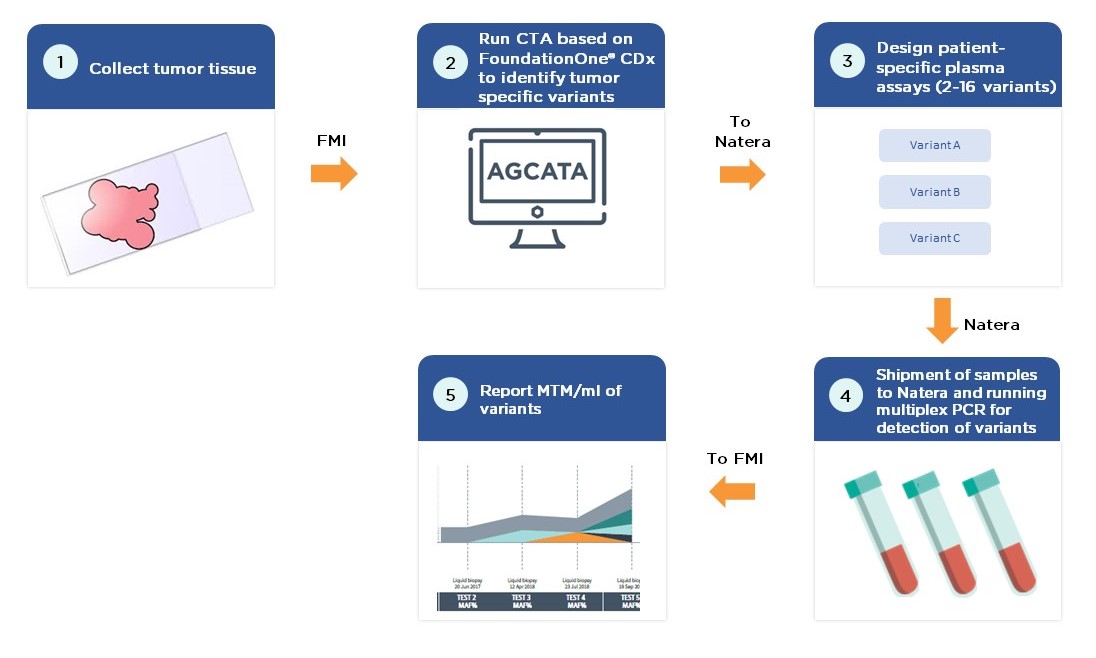
**

**Supplementary Figure S2.**

Consort diagram. Abbreviations: ΔctDNA, change in circulating tumor DNA; ctDNA, circulating tumor DNA; MSI, microsatellite instability; PD-L1, programmed cell death-ligand 1; TIL, tumor-infiltrating lymphocyte; TMB, tumor mutational burden; W4, week 4; W8, week 8.

**Supplementary Figure S3.** Baseline ctDNA level varies by disease type and trends with OS. **A,** Breakdown of availability and ctDNA status of baseline blood specimens by disease. **B,** Distribution of baseline ctDNA levels by disease. **C,** Kaplan-Meier curve of OS for patients with high-shed and low-shed diseases stratified at the median ctDNA level for each disease class. **D,** Relationship between baseline target lesion size and baseline ctDNA level. Scatter plot of Spearman correlation coefficient (*R*) and *P* value are shown. Open circles relate to data points not included in correlation analysis due to nondetection/nonshedding. Abbreviations: CI, confidence interval; CRC, colorectal cancer; ctDNA, circulating tumor DNA; MTM/mL, mean tumor molecules per mL of plasma; OS, overall survival; ref, reference.





**Supplementary Figure S4.** Change in ctDNA from baseline through W8 is strongly correlated with OS. Kaplan-Meier curves of OS for patients stratified by increasing versus decreasing ctDNA from baseline through W8 for (**A**) all patients with an available baseline and on-treatment sample (median OS = 29.21 vs. 11.47 months; *P* = 0.0105; HR = 11.6), (**B**) patients with CRC, and (**C**) mixed tumors. Kaplan-Meier curves for OS stratified by increasing versus decreasing ctDNA from baseline to W8 for all patients with an available W8 specimen (**D**). Abbreviations: CRC, colorectal cancer; ctDNA, circulating tumor DNA; OS, overall survival; W8, week 8.


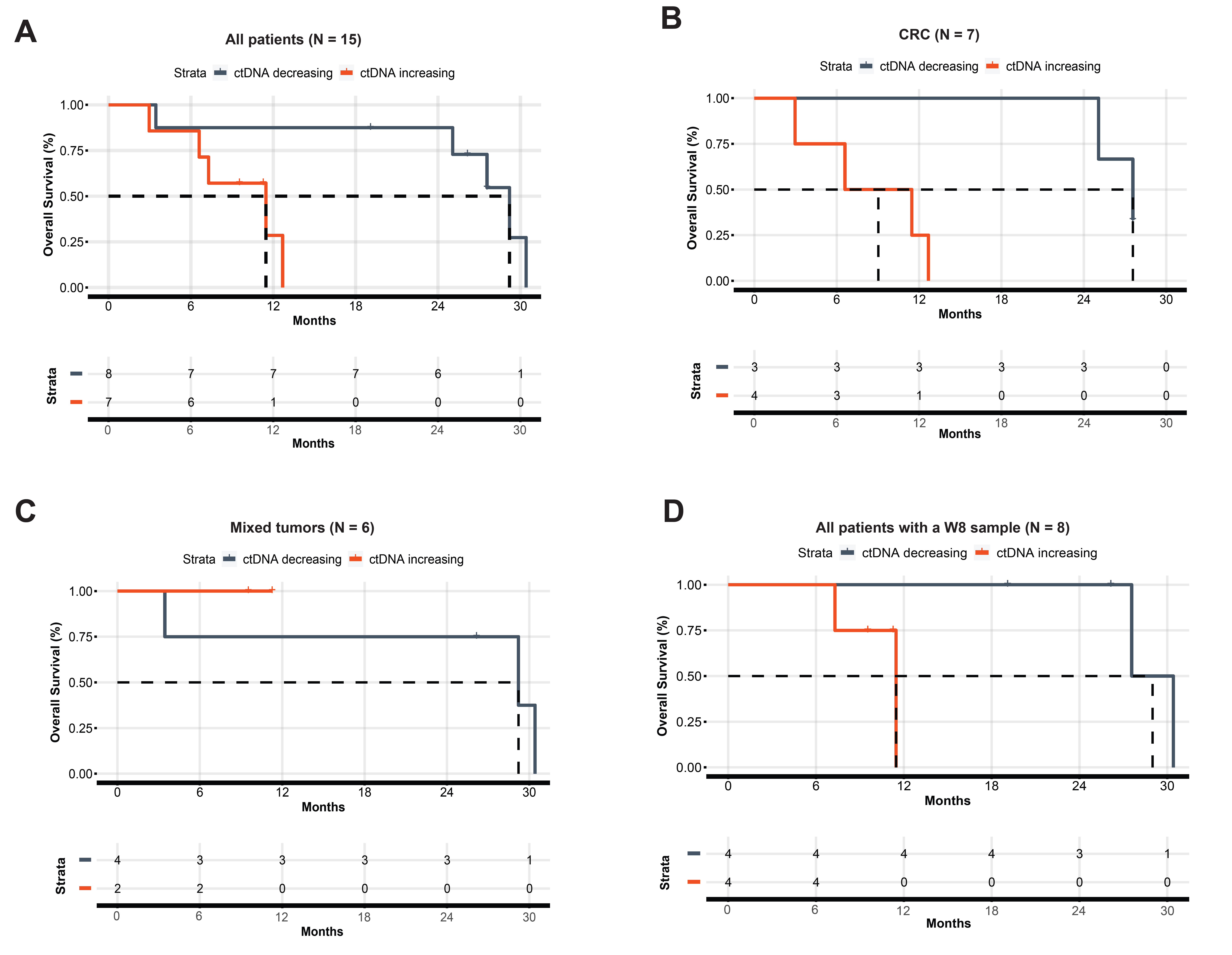


**Supplementary Figure S5.** Change in ctDNA from baseline through W8 is correlated with change in target lesion size and survival. **A,** Waterfall plot of ctDNA change from baseline through W8 and OS for 15 patients with available baseline and on-treatment samples. **B,** Relationship between change in target lesion size versus ctDNA through W8. Scatter plot of Spearman correlation coefficient (*R*) and *P* value are shown. Open circles relate to data points not included in correlation analysis due to nondetection/nonshedding. Abbreviations: ctDNA, circulating tumor DNA; OS, overall survival; W8, week 8.

**Supplementary Figure S6.** Relationship between ctDNA change and clinical response. **A,** Kaplan-Meier curve of OS for patients stratified by their ctDNA change from baseline to W4 and best ORR for 13 patients with available baseline and W4 ctDNA samples. **B,** ctDNA kinetics for patients with a best ORR of CR/PR/SD and PD. **C,** Four patients with a ctDNA increase observed at W4 followed by PD on imaging. Abbreviations: CR, complete response; CT, computed tomography; ctDNA, circulating tumor DNA; ORR, overall response rate; OS, overall survival; PD, progressive disease; PR, partial response; RECIST, Response Evaluation Criteria in Solid Tumours; SD, stable disease; W4, week 4; W8, week 8.


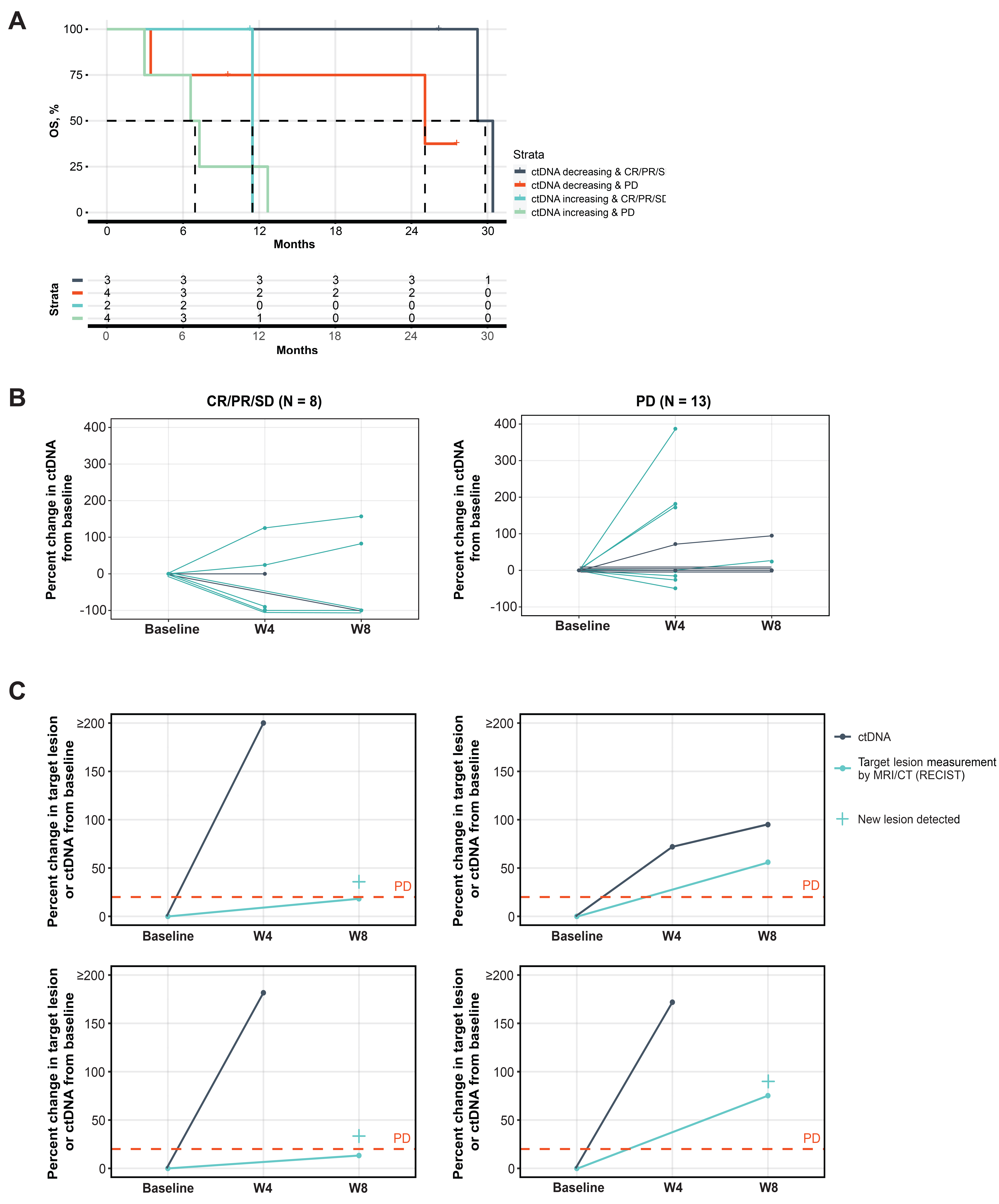

Supplement: Supplementary file 1 — Fig. S1. FoundationOne Tracker workflow diagram. Fig. S2. Consort diagram. Fig. S3. Baseline ctDNA level varies by disease type and trends with OS. Fig. S4. Change in ctDNA from baseline through W8 is strongly correlated with OS. Fig. S5. Change in ctDNA from baseline through W8 is correlated with change in target lesion size and survival. Fig. S6. Relationship between ctDNA change and clinical response. [file MOL2-17-298-s001.docx]
